# Spatio-Temporal Distribution of *Acinetobacter baumannii* in Germany—A Comprehensive Systematic Review of Studies on Resistance Development in Humans (2000–2018)

**DOI:** 10.3390/microorganisms8030375

**Published:** 2020-03-06

**Authors:** Gamal Wareth, Christian Brandt, Lisa D. Sprague, Heinrich Neubauer, Mathias W. Pletz

**Affiliations:** 1 Friedrich-Loeffler-Institut, Institute of Bacterial Infections and Zoonoses, Naumburger Str. 96a, 07743 Jena, Germany; lisa.sprague@fli.de (L.D.S.); heinrich.neubauer@fli.de (H.N.); 2 Faculty of Veterinary Medicine, Benha University, Moshtohor, Toukh 13736, Egypt; 3 Institute for Infectious Diseases and Infection Control, Jena University Hospital, Am Klinikum 1, 07747 Jena, Germany; christian.jena@gmail.com (C.B.); mathias.pletz@med.uni-jena.de (M.W.P.); 4 Research Campus Infectognostics, Philosophenweg 7, 07743 Jena, Germany

**Keywords:** *Acinetobacter baumannii*, resistance development, distribution, Germany, systematic review, human

## Abstract

*Acinetobacter* (*A.*) *baumannii* has gained global notoriety as a significant nosocomial pathogen because it is frequently associated with multi-drug resistance and hospital-based outbreaks. There is a substantial difference in the incidence of *A. baumannii* infections between different countries and within Germany. However, its continuous spread within Germany is a matter of concern. A systematic literature search and analysis of the literature published between 2000 and 2018 on *A. baumannii* in humans was performed. Forty-four studies out of 216 articles met the criteria for inclusion, and were selected and reviewed. The number of published articles is increasing over time gradually. Case reports and outbreak investigations are representing the main body of publications. North Rhine-Westphalia, Hesse and Baden-Wuerttemberg were states with frequent reports. Hospitals in Cologne and Frankfurt were often mentioned as specialized institutions. Multiresistant strains carrying diverse resistance genes were isolated in 13 of the 16 German states. The oxacillinase *bla*OXA-23-like, intrinsic *bla*OXA-51-like, *bla*OXA-58 variant, *bla*NDM-1, *bla*GES-11, *bla*CTX-M and *bla*TEM are the most predominant resistance traits found in German *A. baum*annii isolates. Five clonal lineages IC-2, IC-7, IC-1, IC-4 and IC-6 and six sequence types ST22, ST53, ST195, ST218, ST944/ST78 and ST348/ST2 have been reported. Due to multidrug resistance, colistin, tigecycline, aminoglycosides, fosfomycin, ceftazidime/avibactam and ceftolozan/tazobactam were often reported to be the only effective antibiotics left to treat quadruple multi-resistant Gram-negative (4MRGN) *A. baumannii.* Dissemination and infection rates of *A. baumannii* are on the rise nationwide. Hence, several aspects of resistance development and pathogenesis are not fully understood yet. Increased awareness, extensive study of mechanisms of resistance and development of alternative strategies for treatment are required. One-Health genomic surveillance is needed to understand the dynamics of spread, to identify the main reservoirs and routes of transmission and to develop targeted intervention strategies.

## 1. Introduction

*Acinetobacter* (A.) *baumannii* is a ubiquitous, Gram-negative, non-motile bacterium associated with hospital-acquired infection globally. It has been classified among the most dangerous multiple drug-resistant (MDR) pathogens worldwide. It has been listed in the “priority 1: critical” pathogens list of the World Health Organization (WHO). The pathogen was found in humans, animals, foods and the environment. *A. baumannii* is associated with severe infection, high mortality rates and massive economic loss [1]. Since the late 20th century, this bacterium emerged as a major cause of healthcare-associated infection in critically ill patients causing bloodstream infections, ventilator-associated pneumonia, meningitis, endocarditis, wound infection, urinary tract infections, skin and soft tissues infections [2]. Mortality rates can reach 35% in ventilator-associated pneumonia and bloodstream infections [3]. Members of the genus *Acinetobacter* are usually environmental bacteria. For unclear reasons, *A. baumannii* emerged as an opportunistic nosocomial pathogen. It has established niches for survival in hospitals as well as in primary animal production systems and the environment [4]. The main vital factors contributing to the extravagant dissemination of *A. baumannii* in hospitals are a large variety of potential sources of entry, environmental resilience and its ability to develop resistance to the majority of antibiotics, particularly the ability to acquire resistance to carbapenems [1,5]. *A. baumannii* becomes resistant via reducing membrane permeability, increasing efflux pump activity and production of a wide variety of β-lactamases. It is also able to acquire resistance via mutations and through horizontal gene transfer [6]. *A. baumannii* exhibits high genetic plasticity, which allows the accumulation of the acquired resistance determinants [7]. Resistance in this pathogen is frequently associated with mobile genetic elements (MGEs) that are transferable between bacteria, thus enabling the rapid dissemination of resistance genes between bacteria of different species and creating a reservoir of antimicrobial-resistance (AMR) genes [7].

For several years, the prevalence of infection with MDR *A. baumannii* has been increasing dramatically in the European countries that is, from 15.4% in 2004 to 48.5% in 2014 [8]. In the late 20th century, *A. baumannii* has emerged as a significant nosocomial pathogen in Germany [9,10,11]. Numerous nosocomial outbreaks were reported among inpatients and preterm infants with a considerable mortality rate of up to 24% after bacteremia in intensive care units at a large teaching hospital of Cologne [12]. Since 2000, the number of reported cases and hospital outbreaks was increasing continuously in most German states [13,14,15,16,17]. Isolates with resistance to all β-lactams, including carbapenems [18] and last resort antimicrobial agents such as colistin and tigecycline [19], were reported nationwide and have become a significant cause of worry of the healthcare system. Several studies on *A. baumannii* have been published from different German states but a systematic review on its spatial and temporal distribution is not yet available. Thus, the current report aims at providing a comprehensive, evidence-based assessment of the literature published from 2000-2018 on *A. baumannii* in Germany.

## 2. Materials and Methods

### 2.1. Data Sources and Search Strategy

A comprehensive search in PubMed, Web of Science, Scopus and the catalogue of the German National Library was performed for articles that include the search terms “*Acinetobacter baumannii*” and “Germany” in their title and abstract. All national and international studies discussing *A. baumannii* in Germany published in both English and German language were collected. References cited within these papers were checked to find additional relevant information.

### 2.2. Selection Criteria and Data Extraction

Studies discussing isolation, epidemiology and antimicrobial resistance of *A. baumannii* from 2000 through 2018 were included. Review articles, congress abstracts, case reports, letters, commentaries and editorials were included. Abstract analysis for each publication was done and, if selected, the full text was carefully analyzed. The following keywords were used—*Acinetobacter baumannii* + Germany + human + antimicrobial resistance. Articles describing other *Acinetobacter* spp., *A. baumannii* in non-human sources, novel diagnostics, development of PCR and studies published before the year 2000 were excluded. For each study, following information was extracted from the full text—first author, year of publication, sampling date, location of sampling (city and state), study population, type and source of samples, number of strains and cases, type of study, resistance profiles of the recovered strains including phenotypic testing and detection of resistance genes. If available, the sequence and clonal type of strains were included.

### 2.3. Data Acquisition and Analysis

**Figure 1:**
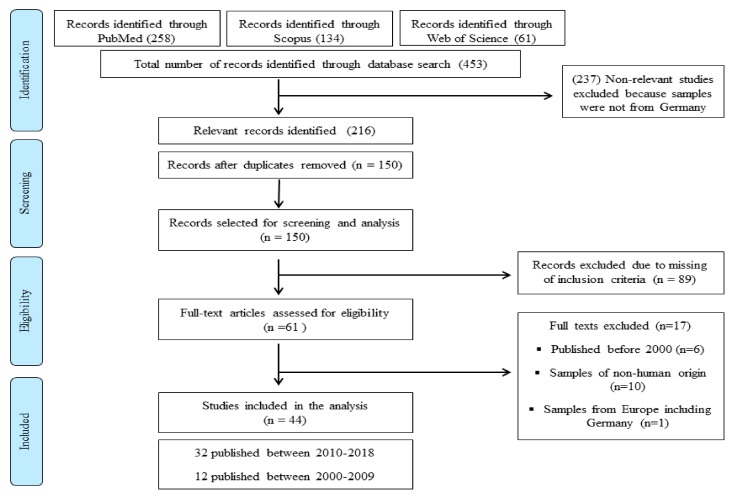
Systematic review flowchart: *Acinetobacter baumannii* in Germany, the flow diagram indicates the inclusion and exclusion of publications at each stage of the systematic review process.

Overall, a total of 216 potentially relevant articles [PubMed (*n* = 114), Scopus (*n* = 69) and Web of Science (*n* = 33)] were identified. Sixty-one articles were selected for screening and forty-four studies were included in the review (Figure 1: PRISMA diagram). The information was categorized starting with the latest one.

### 2.4. Relative Research Interest

The number of publications that are available on PubMed for each year was extracted and the relative research interest was calculated [20]. It is defined as the quotient of specific articles divided by all articles in this year to consider the overall publication growth per year.

## 3. Results

### 3.1. Data Analysis

Case reports represented the majority of the published articles (*n* = 14), followed by outbreak reports (*n* = 7) and evaluation of antibiotic activity (*n* = 6). The remaining studies were surveillance studies, routine microbiological examination and descriptive data analysis. Thirty-seven articles were published in English and seven in German [15,16,17,18,19,20,21]. The sampling date was not reported in 15 studies, while the geographical location of samples was ignored in three studies. Lost information was categorized as “non-determined” and referred to as ‘ND’ in the tables. *A. baumannii* was most often isolated from the bloodstream, wound infections and respiratory and urinary tract infections as well as from skin and rectal swabs. In outbreak investigations it was also isolated from the patient’s environment, medical devices, patient’s associated belongings, room equipment, infusion pumps and oxygen masks [20,22]. In the catalogue of the German National Library, 148 doctoral theses containing the word *Acinetobacter* in their titles were identified. Twenty-one of these were published between 2000–2018. Only nine dissertations with emphasis on isolation, molecular and functional typing, distribution and genetic composition of AMR determinants were found (data are not shown). Three of these were done in Bonn, three in Cologne and one each in Berlin, Freiburg and Frankfurt am Main representing the main centres of *A. baumannii* research.

### 3.2. Temporal Distribution of the Analyzed Studies

**Figure 2:**
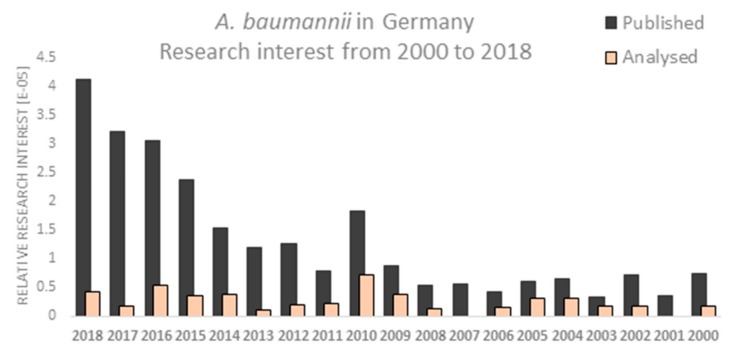
PubMed analysis showing the relative research interest for *A. baumannii* in Germany from 2000–2018. Publications analyzed in this work a marked orange.

The emergence of MDR in *A. baumannii* has been steadily increasing over time and the number of published articles reflects this fact. The relative research interest has been growing after 2013. The year 2010 was super crucial for the research on *A. baumannii* in Germany. PubMed analysis using the search term ‘*A. baumannii* in Germany’ showed a gradual increase in the number of published studies over time with the highest number of annual articles in 2018 (Figure 2). Finally, 44 articles were analyzed. Approximately two thirds of studies (*n* = 32) were published in or after 2010, of them 13 articles have been published in the years 2016–2018 (Table 1).

### 3.3. Spatial Distribution of the Analyzed Studies Based on Geographical Location of Sampling

Most of the published articles are regional studies. Only six studies included samples throughout Germany but without the exact geographical origin of samples. North Rhine-Westphalia, Hesse and Baden-Wuerttemberg contributed eleven, eight and five studies, respectively, followed by Bavaria (four studies), Saxony (three studies) and Berlin and Schleswig-Holstein (two studies each). From Thuringia, Hamburg, Lower Saxony, Saxony-Anhalt, Rhineland-Palatinate and Mecklenburg-Vorpommern, only one study was accessible. Brandenburg, Bremen and Saarland are not present at all. Cologne, the largest city of North Rhine-Westphalia, was the most represented city and was mentioned in six studies. Frankfurt am Main, the largest city of Hesse, was mentioned in five studies. Both cities are centers of *A. baumannii* research. Munich, the largest city of Bavaria, contributed three studies. Leipzig, one of the biggest cities of Saxony, Tuebingen and Freiburg, two cities in Baden-Württemberg, were study areas for two studies each (Table 1).

### 3.4. Official Data Concerning the Infection and Epidemiology of Acinetobacter in Germany

Within the framework of the Infection Protection Act in Germany, the Robert Koch Institute (RKI) is responsible for collecting data on notifiable diseases. The number of nosocomial outbreaks and the number of cases in each outbreak, as well as the number of deaths were recorded for 2010 onwards [59]. As shown in Table 2, no information has been documented in 2010 and 2011 and for the years 2012, 2013 and 2016 the highest numbers of nosocomial outbreaks were reported. The annual statistics on reportable diseases of Germany reported 794 and 784 *Acinetobacter* infections or colonization in 2017 and 2018, with an incidence of 1.0 and 0.9, respectively. North Rhine-Westphalia reported the highest numbers of *Acinetobacter* infections or colonizations with 405 notifications, followed by Bavaria (*n* = 205), Hesse (*n* = 200) and Berlin (*n* = 196) [60]. In contrast, the lowest incidences were reported for the federal states of Bremen (*n* = 6) and Saarland (*n* = 5). It has to be taken into consideration that the numbers mentioned in the RKI statistics do not represent the total number of infections in Germany because only the carbapenem-non-susceptible isolates are reported to the RKI and species of *Acinetobacter* are not differentiated.

### 3.5. Identification of A. baumannii and Antibiotic Susceptibility Testing (AST)

Identification of *A. baumannii* has been done mostly by the VITEK-2 system, MALDI-TOF [18,26] and detection of the intrinsic *bla*OXA-51-like gene [30]. Phenotypic antibiotic sensitivity testing (AST) was performed with the VITEK-2 platform, E-test [28], disk diffusion method [24], antibiotic gradient test and micro-broth dilution using Micronaut-S system [22]. The National Committee for Clinical Laboratory Standards (NCCLS) broth microdilution method and the respective breakpoints were used until 2006 [52]. The broth dilution test was performed following the guidelines of the German Institute for Standardization (DIN) in 2010 [46] and 2013 [39]. The phenotypic detection of resistance in most of the published studies was performed following the Clinical and Laboratory Standards Institute (CLSI) recommendations and the European Committee on Antimicrobial Susceptibility Testing (EUCAST). No breakpoint for tigecycline is approved by EUCAST [49,50]. Thus, the breakpoint of EUCAST for *Enterobacteriaceae* has been used [16].

### 3.6. Resistance Development of A. baumannii 

At the beginning of the 21^st^ century, *A. baumannii* attracted the attention of researchers due to its rapid spread and its resistance to most of the antibiotics used. In a surveillance study performed from 2001 to 2008 in 53 German intensive care units (ICUs), the resistance to imipenem increased from 1.1% in 2001 to 4.5% in 2008 [61]. From 2002 to 2006, the rate of isolation of *A. baumannii* increased in university hospitals of six German federal states (Baden-Wuerttemberg, Hesse, Lower Saxony, Saxony, Schleswig–Holstein and Thuringia) from 2.1% in 2002 to 7.9% in 2006 [46] and the proportion of MDR of *A. baumannii* isolates raised among inpatients [46]. XDR strains resistant to all available antibiotics except colistin have been isolated from wound, skin and respiratory tract samples in an outbreak in 2003 [14]. To overcome the resistance developed against broad-spectrum antibiotics tetracycline, tigecycline a new class of antibiotics derived from tetracycline has been implemented in 2006. Between 2004 and 2007, tigecycline had excellent activity against *A. baumannii* and other Gram-negative nosocomial bacteria [49,50,51]. Until 2005 carbapenems remained the gold standard of therapy for severe *A. baumannii* infections and resistance to carbapenems remained low (0.3%) [54]. In 2006, the first outbreak of carbapenem-resistant *A. baumannii* (CRAb), carrying the *bla*OXA-23-like gene, was reported in Germany. The strain was resistant to imipenem, meropenem, penicillins, cephalosporins, ciprofloxacin, gentamicin, tobramycin and tigecycline and was related to the pan-European *A. baumannii* clone II [16]. Since then, carbapenemase-producing strains have been recovered from several outbreaks every year [26,27,30,33,62]. According to the National Reference Laboratory for Gram-negative nosocomial pathogens at the Ruhr-University Bochum, CRAb was found in 96.3% of strains and *bla*OXA-23 was the most frequent carbapenemase in *A. baumannii* (81.1%) in 2011 [41]. This high resistance rates can be explained by the practice that only non-susceptible strains are sent to the National Reference Laboratory for clarification. It is worth to mention that, the number of cases of carbapenem insensitive *Acinetobacter* infection or colonization was 794 in 2017 and 784 in 2018 [60]. In contrast, the ARS data demonstrate that only 4% of *A. baumannii* ABC complex strains were not carbapenem sensitive in 2015 to 2018 (https://ars.rki.de/Content/Database/ResistanceOverview.aspx).

Susceptibility to tigecycline and colistin was reported in four and nineteen studies, respectively (Table 3). Susceptibility to colistin and tigecycline remained unaffected until December 2013. At this time, the first reported pan drug-resistant (PDR)-CRAb strain was recovered from a patient at Goethe University hospital Frankfurt after a previous hospitalization in Greece. This strain was also resistant to colistin [19]. Until 2018 colistin-resistant strains were detected three times in Germany. One was isolated from a human in 2013 in Frankfurt [19], one from a dog in Giessen in 2011 [63] and one from wastewater in 2017 [64]. It has to be taken in consideration that most of the earlier colistin testing was performed by gradient tests for example, disc diffusion, E-test or with VITEK. EUCAST has revised the recommendation for colistin AST and microdilution technique is considered the only reliable method [65]. Tigecycline resistant strains were reported five times: once in 2016 in Leverkusen [30], once in 2014 from Frankfurt [19], two times in 2010 from the university hospital Frankfurt [47] and from the university hospital Rostock [15] and once in 2009 with undetermined location [16]. Colistin, tigecycline, aminoglycosides, fosfomycin, ceftazidime/avibactam and ceftolozan/tazobactam are often used antibiotics to treat 4MRGN *A. baumannii* infection in Germany [66]. Information regarding the resistance patterns of *A. baumannii* are shown in Table 3. 

### 3.7. Resistance Genes in A. baumannii 

**Figure 3:**
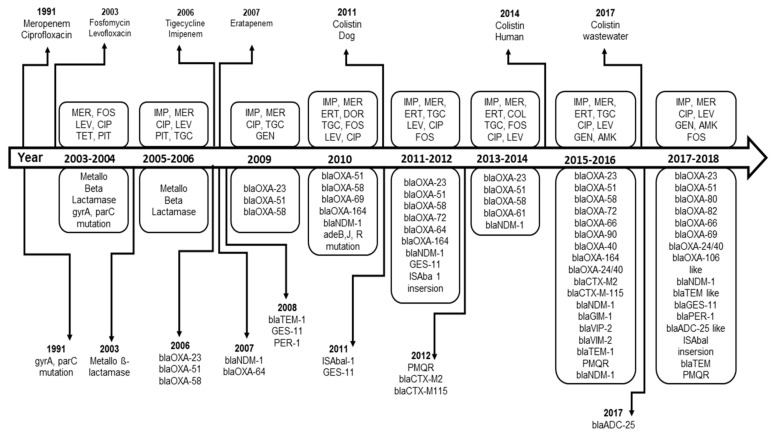
Chronological emergence of ß-lactam resistance in *A. baumannii* in Germany.

Twenty-eight of the selected studies described AMR genes. As shown in Table 3, the metallo-ß-lactamase was first described in studies published in 2004, 2005 and 2006 [14,52,53]. The oxacillinase *blaOXA*-23-like, the intrinsic *bla*OXA-51-like, the New Delhi Metallo-ß-lactamases (*bla*NDM) and the *bla*OXA-58-like variant are the most dominant resistance genes that have been described in *A. baumannii* from Germany (Table 3). The gene *blaOXA*-23-like, the intrinsic *bla*OXA-51-like and the *bla*OXA-58-like variants were found in a CRAb strain for the first time in an outbreak, which occurred at a German university medical center in 2006. However, the location of the sampling was ignored [16]. Those three genes have been reported later on in sixteen, elven and seven studies, respectively. In 2011, the National Reference Laboratory for Gram-negative nosocomial pathogens at Ruhr-University Bochum found that the *bla*OXA-23 was the most frequent carbapenemase in *A. baumannii* [41]. The first isolation of a *bla*NDM-1 strain was reported in 2007 in Frankfurt [47] and then in ten other studies. Additionally, Guiana extended-spectrum β-lactamases (GES-11) [41], Cefotaxime Hydrolyzing Capabilities (CTX-M) β-lactamases [28], Temoneira (TEM) and Plasmid-Mediated Quinolone Resistance (PMQR) [35], German imipenemase (GIM-1), VIP-2 and Verona integron-encoded-metallo-β-lactamase (VIM-2) [32] and *bla*ADC-25-like [18] have been described in *A. baumannii* (Table 3). Figure 3 represents the chronological emergence of resistance to carbapenems, colistin, tigecycline and quinolones antibiotics as well as the resistance genes according to time of sampling or time of reporting if the time of sampling is not available. 

### 3.8. Clonality and Sequence Typing 

Cluster analysis based on Pulsed-field Gel Electrophoresis (PFGE) and rep-PCR typing identified five clonal lineages of *A. baumannii* in Germany, IC-2, IC-7, IC-1, IC-4 and IC-6. The international clone IC-2 is the most common clonal lineage widespread in Germany and was present in seven studies. Followed by IC-7 and IC-1, each was reported in two studies, while IC-4 and IC-6, each was identified only in one study. IC-2 was detected in five studies alone [16,19,27,33,42] and in combination with IC-6 once [28] and with IC-1, IC-7 and IC-4 in another study during the investigation of strains collected from different laboratories in the years 2005, 2007 and 2009 [39]. Only six sequence types of *A. baumannii* have been reported in the reviewed articles. Multilocus sequence typing (MLST) revealed ST22 and ST53 types among 13 strains recovered from an outbreak of seven patients in Rostock [15]. ST195 and ST218 were identified in an outbreak involving ten patients in Leverkusen [30]. ST944/ST78 and ST348/ST2 were determined in three isolates recovered from two patients hospitalized in Bavaria and Hesse [28].

### 3.9. The Risk Factors Associated with A. baumannii Infections

Investigation and outbreak studies, as well as case reports analyzed in this review, have reported multiple risk factors for the emergence and acquisition of *A. baumannii* infections. Firstly, travel, medical tourism or contact with health care systems abroad, as well as cross border transfer of patients from highly endemic countries into Germany, lead to the import and emerging presence of *A. baumannii* in the German hospitals. Between June and December 2015, 143 refugees have been admitted to the University hospital Frankfurt, Hesse. 60.8% were positive for MDR Gram-negative bacteria and CRAb strains harboring *bla*OXA-23 and OXA-24 genes were reported in 1.4% of clinical samples examined [27]. *bla*OXA-23 and *bla*OXA-48, the most frequently reported resistance genes in Germany, were detected with high prevalence among Libyan war casualties admitted to the Northwest Medical Center (NMC) in Frankfurt/Main, Hesse between August 2016 and January 2017 [24]. MDR *A. baumannii* was recovered from patients in German hospitals after previous hospitalization in Russia [28,34], Thailand [30], Serbia [42,47], Poland [43], China, the United Arabic Emirates and Croatia [37], Greece and Italy [53] and other Mediterranean countries [52]. A patient was infected in Cameroon [14]. First reports on PDR (pan-drug resistant) *A. baumannii* appeared in 2001 and an outbreak was reported in Spain in 2002 [67]. Subsequently, the first isolation of a PDR strain in Germany belonging to the IC-2 cluster was done from the skin and rectal swab samples of a patient previously hospitalized in Greece [19]. In contrast, a CRAb strain harboring *bla*OXA-23 was isolated from a hip joint infection in a patient with no history of traveling abroad at the University hospital Bonn [33]. Between 2005 and 2009, 140 CRAb strains were isolated in 15 different medical centers in Germany, only 32% of these strains belonged to one of the known international clonal lineages [39]. 

Secondly, during a (long-term) ICU stay, *A. baumannii* can be acquired via mechanical ventilation. An epidemic strain of *A. baumannii* was transferred from the index patient to other nine patients at the same hospital in Leverkusen, North Rhine-Westphalia within 16 days [30]. Cross-transmission via the hands of health care workers from colonized or infected patients has been described in an epidemic setting in a German university medical center in Berlin [16]. In one year, 44 individual patients acquired infection in a hospital in Cologne. *A. baumannii* was recovered from the washbasin and fixation bath [18], from medical devices, infusion pumps and oxygen masks [15,16]. An identical macrorestriction pattern (PFGE-type) was detected in isolates of two different hospitals in 2008 in North Rhine-Westphalia confirming that inter-hospital transmission is possible [26]. Moreover, a prolonged hospital stay is frequently associated with exposure to antibiotics [68]. *A. baumannii* can rapidly acquire resistance by mutation during antimicrobial therapy [44]. Selective pressure due to preceding antimicrobial treatment was a significant factor in acquiring carbapenem-resistance in a study from Hamburg-Eppendorf, Hamburg [21]. 

Finally, the environmental resilience of *A. baumannii* and environmental contamination facilitates the spread and dissemination of infection from colonized or infected patients. Examination of environmental samples during an outbreak revealed relevant dissemination of *A. baumannii* [18]. *A. baumannii* was isolated from the patients’ environment, patients’ belongings and room equipment [15,16]. *A. baumannii* can survive in the environment for a prolonged time and it can colonize non-human hosts for example, pet animals with ease [69]. The occurrence of genotypically related clones I–III in animals and humans in Germany raises the concern about the possibility of a spillover of the organisms from humans to animals or *vice versa* [63]. Strains harboring resistance genes coding for clinically relevant antibiotics can enter the clinical setting via food and community routes [70,71]. Thus, humans can be colonized by *A. baumannii* through the food chain or from other environmental sources for example, wastewater, hatchery system and dust.

## 4. Discussion

*Acinetobacter baumannii* is one of the most threatening emerging environmental pathogens, causing particularly nosocomial infections in humans in Germany. The number of published studies concerning *A. baumannii* is dramatically increasing worldwide and also in Germany. This systematic review investigated the spatial and temporal distribution of *A. baumannii* in humans in Germany based on published data from 2000 to 2018. The pathogen was extensively studied in the last few years. This increase of interest reflects the increasing clinical impact and its ability to acquire resistance against the majority of antibiotics. *A. baumannii* MDR strains that is, strains with resistance to all β-lactams including carbapenems and production of β-lactamases of several types, were reported nationwide. A prominent number of published articles originates from North Rhine-Westphalia. This state is located in the West of Germany and has the highest population of German states. A considerable number of reports also describe the situation of Hesse, which is a central German state. The cities Cologne and Frankfurt-am-Main are centres of *A. baumannii* research and are home to tertiary care clinics and hospitals which treat a relatively high numbers of patients in ICUs. These hospitals also run large highly specialized treatment units that received patients from abroad [18,24]. The obtained results are in harmony with the official data of the Robert Koch Institute (RKI) considering numbers of infection or colonization of carbapenem non-susceptible *Acinetobacter* spp. and incidence [60]. The official data published by RKI were included to avoid loss of information, aware the shortcoming of these data that is, no differentiation between the different species was made and that numbers do not present the total number of infections in Germany, as only carbapenem non-susceptible strains have to be notified. Information published by the ARS project cover data for the whole *A. baumannii* complex group. 

The identification of *A. baumannii* was mostly based on the VITEK-2 system, MALDI-TOF and PCR. The failure of the semi-automated VITEK-2 system to correctly identify *A. baumannii* and predict carbapenem susceptibility is well known [44]. MALDI-TOF log score values >2000 were accepted in some laboratories for species identification [72]. However, evaluation of species-specific score cut-off values of clinical *A. baumannii* revealed that a score value > 2.3 is the valid score value for species identification [73]. Species misidentification and false AST may negatively influence clinical outcome. *A. baumannii* is part of the ACB complex and it is difficult to distinguish *A. baumannii* from other species phenotypically. A proper, accurate and reliable molecular identification of *A. baumannii* to species level is needed and a typing method with high discriminatory power is crucial [18,44] for diagnosis, epidemiological trace back and vaccine production. Rates of colonization and infection with triple (3MRGN) and quadruple (4MRGN) *A. baumannii* are on the rise in Germany [17,29,36]. However, this type of classification is specific for Germany only. Carbapenems are the gold standard to date for the treatment of severe infections with MDR strains. However, the appearance of CRAb poses new challenges for treatment strategies [66]. Nevertheless, colistin and tigecycline are therapeutic options and showed efficacy in the treatment of MDR strains [15,25,37,51]. The appearance of colistin and tigecycline resistant [47] and colistin-resistant strains carrying the *mcr-*1 gene in non-human sources [64] will result in limited effectiveness in the near future. Several methods have been used to detect colistin and tigecycline resistant strains. The lack of EUCAST and CLSI breakpoints for tigecycline hampers the detection of tigecycline resistance and is a big challenge for clinicians [74]. Accordingly, the breakpoint for *Enterobacteriaceae* was recommended and used in different studies in the past [16]. However, caution is needed during interpretation. *A. baumannii* is intrinsically resistant to fosfomycin [75]. Only six articles were found discussing resistance to fosfomycin. The first fosfomycin resistant *A. baumannii* isolate was reported from an outbreak at the university hospital Tuebingen in 2004 [14] and then MDR strains resistant to fosfomycin were reported again in 2010, 2011, 2014 and 2017 [19,25,43,44]. According to the Surveillance of Antibiotic Use and Resistance in Intensive Care Units (SARI-ICUs) project, that was initiated in Germany in 2000, the resistance rate of MRGN pathogens increased markedly from 2001 to 2015 [76]. The increase in *A. baumannii* isolates with resistance to imipenem has been reported from 2005 onwards with a pronounced trend. The resistance rate has more than doubled over recent years and reach to 43% in 2015 [76]. The antibiotic resistance surveillance (ARS) project revealed that, the level of resistance in *A. baumannii* isolates obtained from patients in ICUs is about three times that higher than of isolates obtained from patients in normal and outpatient settings. 7.6% of *A. baumannii* strains were carbapenem resistant in 2014 and 4% in 2017 and 2018. However, it has to be kept in mind that this information is not restricted to *A. baumannii* but include all of the *A. baumannii* complex group (https://ars.rki.de/Content/Database/ResistanceOverview.aspx). 

The *bla*OXA-23, *bla*OXA-51-like and *blaOXA*-58-like, as well as *bla*NDM-1 were the resistance genes most often found in *A. baumannii* in the last ten years followed by *bla*GES-11, *bla*CTX-M and *bla*VIM. Since the first description of the *bla*OXA-23 in a clinical isolate of *A. baumannii* in Scotland in 1995 [77], *A. baumannii* harboring the *bla*OXA-23 gene spread to hospitals worldwide and were also detected in companion animals, livestock, environment and ectoparasites [78]. It has become the most frequent carbapenemase gene among *A. baumannii* in most countries. Transfer of resistance genes from bacteria of other genera is a predominant mechanism of acquiring resistance to carbapenems in *A. baumannii.* The *bla*NDM-1 was identified mainly in *E. coli* and *K. pneumoniae* and to a lower extent in *Pseudomonas* and *Acinetobacter.* The first occurrence of *A. baumannii* carrying *bla*NDM-1 in Germany dates bake to the year 2007 [47,48]. Hence, current PCR protocols only identify resistance-coding genes but provide no in-depth information regarding the functions and mechanisms of resistance development or actual clinical resistance. Detection and characterization of AMR genes are gradually moving from PCR to high throughput identification via sequencing and *in-silico* detection utilizing several databases now. However, the use of next-generation sequencing (NGS) to investigate AMR in *A. baumannii* is still rare and has just begun to start in Germany.

This discriminatory power enables transmission-chain analysis, optimizes surveillance and promotes suitable containment measures. It also offers the traits to identify transmission dynamics [79]. Molecular characterization of AMR in *A. baumannii* often has been performed by using conventional methods and very few studies utilizing the WGS technology have been performed [18,31,35]. For outbreak investigation, PFGE has been considered the gold standard for a long time but it has been replaced by NGS technology due to its higher discriminatory properties. Most of the WGS based studies on *A. baumannii* were performed in outbreak settings and there is little data on WGS in the context of surveillance.

International travel of colonized patients, especially from countries with high prevalence for example, Mediterranean and Asian countries has resulted in and still resulting in the introduction and later spread of MDR *A. baumannii* to Europe and subsequently into Germany [53]. Following a fatal outbreak of six patients with *A. baumannii* infection-related death at a tertiary care facility in the northwest United States, comparative genomics of strains showed a close relation to strains isolated from Germany in 2003 [80]. Isolates from Germany were found to share the same rep-PCR pattern with isolates from Turkey and the USA [81] and identical to those from isolates from the UK [82]. Thus, cross border transmission of MDR *A. baumannii* or its resistance genes have to be considered and the colonization status of patients treated abroad should be handled with care to avoid the spread of this pathogen into countries with low incidence rates [53]. WGS can be the tool of choice to assist public health officer and clinicians to contain spread and to initiate well timed start of treatment with the best medication.

## 5. Conclusions

In conclusion, the published knowledge on the existence and general distribution of *A. baumannii* in the German population is gradually increasing over time but is mainly based on regional studies, outbreak investigations and case reports. Only few countrywide studies have been done yet. Several aspects of resistance development remain cryptic. Large cities with tertiary care hospitals and vital tourism are hot spots for research and publication activity on *A. baumannii.* MDR strains harboring diverse resistance genes were found in most of the federal states. Five clonal lineages and six sequence types of *A. baumannii* have been identified within the last 18 years in Germany. *A. baumannii* is part of the ACB complex and it is difficult to distinguish it from other species. Due to clinical needs, its differentiation from other members of the ACB complex species, particularly the closely related *A. pittii* and *A. nosocomialis* is required. Accurate and safe diagnostics for species identification as well as the definition of breakpoints for all antibiotics used needs to be established. Several risk factors contributing to the spread and dissemination of infection have been identified for example, travelling, medical tourisms, long-term stay at ICUs and the environmental contamination. Dissemination and infection rates of MDR (particularly those known in Germany as 3MRGN-Ab and 4MRGN-Ab) are on the rise nationwide and need to be considered a threat to public health. 

Due to the nature of the data reported to RKI they cannot differentiate between the different species causing infection or colonization. The ARS project shaves this limitation as it collects data on the *A. baumannii* complex following a holistic approach. The reported data published by the National Reference Laboratory and the RKI do not sum up the total number of infections or colonizations in Germany because only carbapenem non-susceptible *Acinetobacter* strains need to be reported to the NRL and RKI. The nationwide One-Health genomic surveillance of *A. baumannii* using NGS technology to understand the dynamics of resistance and mobile genetic elements (MGEs) has to be in the center of public efforts, now the NGS is afford and available. Especially pets, livestock, food and the environment are neglected reservoirs and isolates from theses niches need to be investigated to assess their impact on human health and economy. Thus, identification of novel and alternative strategies for treatment, collaboration between the veterinary and human health sectors and the increase of awareness in the public and in the risk group are fields identified to be improved.

## Figures and Tables

**Table 1:** Spatio-temporal distribution of studies on *A. baumannii* of human origin in Germany from 2000 to 2018.

Ref	First Author	Yr. of Report	Sampling Date	Location of Sampling	Study Population	Sample Type	No. Of Strains/Cases	Type of Study
[21]	(Katchanov et al., 2018)	2018	Sep. 2015 till Aug. 2016	Hamburg-Eppendorf, Hamburg	119 Individual patients	Pneumonia and bloodstream infection	18 patients	Retrospective analysis
[18]	(Wendel et al., 2018)	2018	Apr. 2017 till Mar. 2018	Cologne, North Rhine-Westphalia	44 Individual patients	Rectal and nose/throat swabs, tracheal secretion and patients environment	43/48 isolates	Retrospective analysis
[22]	(Kerz et al., 2018)	2018	ND	Mainz, Rhineland-Palatinate	One patient	Blood culture and CSF	1 case	Case report
[23]	(Papan et al., 2018)	2018	Feb. till Dec. 2015	Munich, Bavaria	29 Individual patients	Wound, skin abscess, burn, synovial and thoracic drainage	1 isolate	Routine bacterial diagnosis
[24]	(Lohr et al., 2018)	2018	Aug. 2016 till Jan. 2017	NMC, Frankfurt/Main, Hesse	67 Libyan war victims	Rectal, nasopharyngeal and inguinal swabs and wounds	15 isolates	Prevalence and investigation
[25]	(Tafelski et al., 2017)	2017	ND	Berlin, Berlin	1 patient	Cranial wound	1 isolate	Case report
[26]	(Rieber et al., 2017)	2017	Feb. 2008 till Jan. 2012	North Rhine-Westphalia	22/44 hospitals	Respiratory tract, wound, urine, vascular, joint, catheter, blood, intraoperative tissue, pharyngeal swabs	79/1167 isolates	Investigation study
[27]	(Reinheimer et al., 2016)	2016	Jun. till Dec. 2015	UHF, Frankfurt/Main, Hesse	143 refugee, 1489 residents	Rectal swabs	4 isolates	Investigation study
[28]	(Pfeifer et al., 2016)	2016	Jul. 2012 till Sep. 2013	Hospitals in Hesse and Bavaria	2 Russian patients	Intraoperative wound swab	3 isolates	Case report
[29]	(Huenges et al., 2016)	2016	ND	Kiel, Schleswig-Holstein	1 patient	Skin swab and urine	1 case	Case report
[30]	(Molter et al., 2016)	2016	7 Dec. 2011 till 24 Jan. 2012	Leverkusen	Outbreak, 10 patients	Throat, groin, trachea	10 cases	Outbreak investigation
[31]	(Willems et al., 2016)	2016	Oct. 2013 till Mar. 2014	Muenster, North Rhine-Westphalia	2 ICUs at UH	Skin, central intravenous catheter, respiratory samples	32 isolates	Routine surveillance study
[32]	(Ghebremedhin et al., 2016)	2016	ND	UH Bochum and Wuppertal, North Rhine-Westphalia	Strain collection	Culture isolates from laboratories	38 isolates	Diagnostic study
[33]	(Hischebeth et al., 2015)	2015	ND	UH Bonn, North Rhine-Westphalia	1 patient	Hip joint infection	1 isolate	Case report
[34]	(Dersch et al., 2015)	2015	ND	Freiburg, Baden-Württemberg	1 patient	Catheter associated ventriculitis/CSF	1 isolate	Case report
[35]	(Leistner et al., 2015)	2015	Jan. 2012 till Dec. 2013	Berlin, Berlin	213 Libyan patients	Rectal and nasal swabs	4 cases	Prevalence and investigation
[36]	(Hauri et al., 2015)	2015	Jan. 2012 till Sep. 2014	Hesse	Laboratories information	Notification cases	185 complex	Data survey
[17]	(Siemers et al., 2014)	2014	24 Dec. 2012 till 16 Jan. 2013	Burn center in Halle (Saale), Saxony-Anhalt	Outbreak, 7 patients	Nasal and throat swabs, blood, skin and wounds	7 cases	Outbreak investigation
[19]	(Gottig et al., 2014)	2014	Dec. 2013	Frankfurt, Hesse	1 patient	Skin and rectal swab	1 PDR strain	Case report
[37]	(Lahmer et al., 2014)	2014	ND	Munich, Bavaria	Outbreak, 6 patients	Blood culture, bronchoalveolar lavage and ascites	6 cases	Outbreak investigation
[38]	(Kleinkauf et al., 2014)	2014	Apr. 2012 till Mar. 2013	Frankfurt/Main, Hesse	Health care facilities	Notification cases, mainly wound swabs	28 cases	Descriptive analysis of data
[39]	(Schleicher et al., 2013)	2013	2005, 2007 and 2009	ND	15 Diagnostic laboratories	Respiratory tract, wound, intra-abdominal, blood culture	140 isolates	Surveillance study
[40]	(Kaase, 2012a)	2012	2011	multi-centric study, NRL for MDRGNB, Bochum	Strain collection	Respiratory, wound, urine and blood culture	292 isolates	Surveillance study
[41]	(Kaase, 2012b)	2012	2011	Nationwide (16 States), NRL for MDRGNB, Bochum	Strain collection	ND	287 isolates	Surveillance study
[42]	(Pfeifer et al., 2011)	2011	2007	ND	1 patient	ND	1 isolate 161/07	Investigation study
[43]	(Wagner et al., 2011)	2011	ND	Leipzig, Saxony	1 patient	Deep wound	1 isolate	Case report
[44]	(Higgins et al., 2010)	2010	ND	Cologne UH, North Rhine-Westphalia	1 patient	Respiratory, blood and bedsore	33 isolates	Case report
[45]	(Aivazova et al., 2010)	2010	ND	LMU, Munich, Bavaria	1 patient	Vaginal smear	1 isolate	Case report
[46]	(Wadl et al., 2010)	2010	2002 till 2006	Thuringia, Baden-Wuerttemberg, Hesse, Lower Saxony, Saxony, Schleswig– Holstein	4 from 6 University hospitals	Respiratory, blood, urine, non-specific	1190 isolates	Retrospective and descriptive analysis of data
[47]	(Gottig et al., 2010)	2010	2007	UHF, Frankfurt, Hesse	1 patient	Skin, tracheal secretion, vessels prosthesis and wound	ND	Case report
[15]	(Frickmann et al., 2010)	2010	ND	UH Rostock, Mecklenburg-Vorpommern	Outbreak, 7 patients	Medical devices, patient associated objectives, room equipment, urine, blood, testicular smear, lower respiratory, buccal swab, skin lesions	13 isolates	Outbreak investigation
[48]	(Pfeifer, 2010)	2010	2007	South-Western Germany	Hospital	ND	Ten isolates	Case report
[49]	(Seifert and Dowzicky, 2009)	2009	2004–2007	Throug out Germany	13 Centers	ND	187 isolates	Evaluation of antibiotic activities
[50]	(Kresken et al., 2009)	2009	2007	Throug out Germany	15 Centers	Peritoneal cavity, respiratory, blood, urine and wound	117 isolates	Evaluation of antibiotic activities
[16]	(Kohlenberg et al., 2009)	2009	18 Jun till 31 Aug. 2006	ND	Outbreak, 39 patients	Wound, lower respiratory (one from each infusion pump, oxygen mask, hand of health care worker)	39 isolates	Outbreak investigation
[51]	(Rodloff et al., 2008)	2008	Jan. 2004 till Aug. 2006	Throug out Germany	7 Medical centers	Blood, wound, skin, urine, respiratory	86 isolates	Evaluation of antibiotic activities
[52]	(Weyrich et al., 2006)	2006	ND	UH, Tuebingen, Baden-Württemberg	1 patient	Blood culture	1 case	Case report
[53]	(Schulte et al., 2005)	2005	ND	South-Western Germany	2 patients	Blood culture, excised tissue from fracture patella	2 isolates	Case report
[54]	(Brauers et al., 2005)	2005	ND	Throug out Germany + HU in Cologne, Freiburg, Leipzig	13 Laboratories	Blood culture, respiratory and urine	395 isolates	Evaluation of antibiotic activities
[14]	(Borgmann et al., 2004)	2004	Feb. until Mar. 2003	UH, Tuebingen, Baden-Württemberg	Outbreak, 14 patients	Wound, skin, respiratory	250 isolates	Outbreak investigation
[55]	(Higgins et al., 2004b)	2004	1991–1992	Cologne, North Rhine-Westphalia	2 Hospital outbreaks	Urine, blood culture	34 cases	Outbreak investigation
[56]	(Frank et al., 2003)	2003	ND	Throug out Germany	19 Laboratories	Urinary, respiratory tract, wound, blood	51 isolates	Evaluation of antibiotic activities
[57]	(Andermahr et al., 2002)	2002	Jun. 1996 till Jun 2001	Cologne, North Rhine-Westphalia	266 patients	Tracheal aspirate	41 cases	Routine bacterial diagnosis
[58]	(Heinemann et al., 2000)	2000	Jul. 1990 till Dec. 1998	Cologne, North Rhine-Westphalia	Outbreaks and sporadic cases	Blood, urine, wound, tracheal secretion	47 isolates	Evaluation of antibiotic activities

ND: not determined, MDR: Multidrug-resistant, MIC: minimum inhibitory concentrations, UH: university hospital.

**Table 2:** Number of the nosocomial outbreaks of carbapenem-non-susceptible *Acinetobacter* spp. in Germany based on official data published by the Robert Koch Institute (RKI) (2010–2018).

Year of Report	No. of Outbreaks	No. of Cases	No. of Deaths	No. of Infection	Incidence
**2018**	5	18	5	784	0.9
**2017**	7	41	11	794	1.0
**2016**	10	38	8	-	-
**2015**	8	56	27	-	-
**2014**	6	29	1	-	-
**2013**	14	67	4	-	-
**2012**	13	86	9	-	-
**2011**	-	-	-	-	-
**2010**	-	-	-	-	-

**Table 3:** Temporal distribution of resistance profiles of *A. baumannii* strains from Germany based on studies published from 2000 to 2018.

No.	References	Sampling Date	Resistant Antibiotics	Susceptible Antibiotics	Resistance Genes	ST/IC
[21]	(Katchanov et al., 2018)	Sep. 2015 till Aug. 2016	MDR, carbapenem-resistant	Colistin in all tested ten isolates	Ten isolates carry *bla*OXA-23	ND
[18]	(Wendel et al., 2018)	Apr. 2017 till Mar. 2018	Two strains were carbapenem-resistant	Carbapenem-2.1. Historical glance on the situation in human	Cephalosporinase-encoding *bla*ADC-25-like, *bla*OXA-106-like, *bla*OXA-51	ND
[22]	(Kerz et al., 2018)	ND	Carbapenemase-producing (Meropenem > 32 mg/L)	Colistin < 1 mg/L	*bla*OXA-23 and *bla*NDM-1	ND
[23]	(Papan et al., 2018)	Feb. till Dec. 2015	One strain was carbapenem-resistant	ND	*bla*OXA-24/40	ND
[24]	(Lohr et al., 2018)	Aug. 2016 till Jan. 2017	Carbapenemase-producing	ND	*bla*OXA-23 (*n* = 9), *bla*NDM (*n* = 2), *bla*NDM+OXA-51-ISAbaI (*n* = 4), TEM and PMQR gene *aac*(6`)-Ib-cr	ND
[25]	(Tafelski et al., 2017)	ND	MDR, imipenem, meropenem ceftriaxone, amikacin, cotrimoxazole, fosfomycin, ciprofloxacin, levofloxacin.	Colistin < 0.5 mg/L and tobramycin	ND	ND
[26]	(Rieber et al., 2017)	Feb. 2008 till Jan. 2012	ESBL, Carbapenem-resistant, ciprofloxacin and gentamicin	Polymyxin B	*bla*OXA-23 (*n* = 66), *bla*OXA-80 (*n* = 1), *bla*OXA-82 (*n* = 3), TEM-like (n = 62), GES-11 (*n* = 1), PER-1 (*n* = 1); *bla*OXA-51-like (OXA-51, OXA-66, OXA-69)	ND
[27]	(Reinheimer et al., 2016)	Jun. till Dec. 2015	Three strains were carbapenem-resistant from refugee and one from residents	ND	*bla*OXA-23 and *bla*OXA-24 in refugee and *bla*OXA-51 in resident	IC-2
[28]	(Pfeifer et al., 2016)	Jul. 2012 till Sep. 2013	Penicillin/inhibitor combinations, cefepime, imipenem, meropenem, aminoglycosides (gentamicin, tobramycin and amikacin), fluoroquinolones (ciprofloxacin) and sulfamethoxazole/trimethoprim.	Colistin	*bla*OXA-40/24 related *bla*OXA-72, *bla*OXA-66, *bla*OXA-90, ESBL-*bla*CTX-M-2-like genes and *bla*CTX-M-115	IC-2, IC-6, ST944/ST78 ST348/ST2
[29]	(Huenges et al., 2016)	ND	4MRGN-ab, intermediate to carbapenems	Colistin	ND	ND
[30]	(Molter et al., 2016)	7 Dec. 2011 till 24 Jan. 2012	Carbapenems, ciprofloxacin and tobramycin; variable to tigecycline (≤0.5 to 4 mg/L) and amikacin (4 to ≥ 64 mg/L).	Colistin (MIC 0.5–1 mg/L)	*bla*OXA-51-like and *bla*OXA-23-like	IC-2, ST195 and ST218
[31]	(Willems et al., 2016)	Oct. 2013 till Mar. 2014	MDR, piperacillin, 3rd/4th generation cephalosporins and fluoroquinolones	Carbapenems, imipenem and meropenem	ND	ND
[32]	(Ghebremedhin et al., 2016)	ND	Carbapenemase-producing	ND	*bla*OXA-23, *bla*OXA-40, *bla*OXA-58, *bla*OXA-72, *bla*OXA-164, *bla*NDM-1, GIM-1, VIP-2 and VIM-2	ND
[33]	(Hischebeth et al., 2015)	ND	Carbapenemase-producing, meropenem (MIC ≥ 32 mg/L) and imipenem (MIC ≥ 32 mg/L), tobramycin (MIC ≥ 16 mg/L) gentamicin (MIC ≥ 16 mg/L)	Colistin only (MIC ≤ 0.5 mg/L)	*bla*OXA-23-like and *bla*OXA-51-like.	IC-2
[34]	(Dersch et al., 2015)	ND	MDR	Colistin	ND	ND
[35]	(Leistner et al., 2015)	Jan. 2012 till Dec. 2013	ESBL, Carbapenem-resistant, Imipenem and Meropenem (MIC ≥ 16 mg/L), Ertapenem (MIC ≥ 32 mg/L).	ND	*bla*OXA-23, TEM-1, GES-11; PMQR genes (*aac*(6´)1b-cr)	ND
[36]	(Hauri et al., 2015)	Jan. 2012 till Sep. 2014	4MRGN, acylureidopenicillins, 3rd/4th generation cephalosporins, carbapenems, fluoroquinolone.	ND	*bla*OXA-23 and *bla*NDM	ND
[17]	(Siemers et al., 2014)	24 Dec. till 2012 to 16 Jan. 2013	4MRGN, acylureidopenicillins, 3rd/4th generation cephalosporins, carbapenems, fluoroquinolone.	Colistin	*bla*OXA-58	ND
[19]	(Gottig et al., 2014)	Dec. 2013	PDR, penicillins/β-lactamase inhibitors, extended-spectrum cephalosporins, carbapenems, tetracyclines, fluoroquinolones, aminoglycosides, folate pathway inhibitors and polymyxins, including colistin (MIC > 128 mg/L) and tigecycline (MIC > 8 mg/L), chloramphenicol and fosfomycin	Intermediately susceptible to minocycline (MIC 8 mg/L)	*Bla*OXA-23, *bla*OXA-51-like (*bla*OXA-61)	IC-2
[37]	(Lahmer et al., 2014)	ND	MDR, Carbapenem-resistant	Tigecycline and colistin	ND	ND
[38]	(Kleinkauf et al., 2014)	Apr. 2012 till Mar. 2013	Carbapenem-resistant		*bla*OXA-23-like and *bla*NDM-1	ND
[39]	(Schleicher et al., 2013)	2005, 2007 and 2009	Non-susceptible to carbapenem and imipenem	ND	*bla*OXA-58-like and *bla*OXA-23-like.	IC-2, IC-1, IC-4, IC-7
[40]	(Kaase, 2012a)	2011	Carbapenem-resistant, imipenem and meropenem,	ND	*bla*OXA-23, *bla*OXA-72, *bla*OXA-58, *bla*OXA-164, NDM-1, ISAba1 insertion *bla*OXA-51 and GES-11	ND
[41]	(Kaase, 2012b)	2011	Carbapenem-resistant, imipenem and meropenem,	ND	*bla*OXA-23, b*la*OXA-72, *bla*OXA-58, *bla*OXA-164, NDM-1, ISAba1 insertion *bla*OXA-51 and GES-11	ND
[42]	(Pfeifer et al., 2011)	2007	MDR, imipenem and meropenem, ertapenem (MIC >32 mg/L), tigecycline (MIC1 mg/L)	Colistin (MIC 0.38 mg/L)	*bla*NDM-1 and *bla*OXA-64	IC-7
[43]	(Wagner et al., 2011)	ND	MDR, ampicillin + sulbactam, piperacillin + tazobactam, cefuroxime, cefotaxime, ceftriaxone, ceftazidime, aztreonam, ertapenem, amikacin, fosfomycin, levofloxacin, ciprofloxacin and moxifloxacin.	Imipenem and tobramycin	ND	ND
[44]	(Higgins et al., 2010)	ND	Cefepime, cefotaxime, cefoxitin, ceftazidime, ciprofloxacin, moxifloxacin, fosfomycin, gentamicin, piperacillin, piperacillin-tazobactam and ampicillin-sulbactam, imipenem, meropenem. Amikacin variable (MIC ≤ 2 and ≥ 32 mg/L), tigecycline variable (MIC 2 and ≥ 8 µg/mL).	Colistin (MIC ≤1 µg/mL) and tobramycin (MIC ≤ 1 µg/mL)	*bla*OXA-51-like (*bla*OXA-69), *bla*OXA-58-like variant (novel *bla*OXA-164 and *bla*OXA-58). *ade*B, *ade*J, novel *ade*R mutation	IC-1
[45]	(Aivazova et al., 2010)	ND	ND	Meropenem	ND	ND
[46]	(Wadl et al., 2010)	2002 till 2006	66 are MDR, beta-lactams (piperacillin/ tazobactam), fluoroquinolones (ciprofloxacin, levofloxacin), cephalosporins (ceftazidime, cefepime), Aminoglycosides (tobramycin, gentamicin) and trimethoprim/ sulphamethoxazole combination cotrimoxazole.	Lowest resistance in carbapenems (meropenem, imipenem) and beta-lactams (ampicillin/sulbactam).	ND	ND
[47]	(Gottig et al., 2010)	2007	Carbapenems, fluoroquinolones, aminoglycosides, tigecycline, aztreonam,	Colistin (MIC ≤ 1 µg/mL)	*bla*NDM-1	ND
[15]	(Frickmann et al., 2010)	ND	MDR (ampicillin-sulbactam, piperacillin-tazobactam, cefuroxime, cefotaxime, cefpodoxime, ceftazidime, imipenem, meropenem, ciprofloxacin, levofloxacin, gentamycin, tobramycin, tetracycline, trimethoprim-sulfamethoxazole, tigecycline, doripenem, cefepime, ertapenem, moxifloxacin, fosfomycin, aztreonam).	Colistin	ND	ST22, ST53
[48]	(Pfeifer, 2010)	2007	MDR, carbapenems	Colistin (MIC 0.25 µg/mL)	*bla*NDM-1	ND
[49]	(Seifert and Dowzicky, 2009)	2004–2007	Ampicillin and amoxicillin/clavulanic acid (MIC ≥ 64 µg/mL), ceftriaxone (MIC ≥ 128 µg/mL) and cefepime (MIC 32 µg/mL)	Tigecycline, amikacin and minocycline (MIC ≤1 µg/mL), imipenem, meropenem, levofloxacin,	ND	ND
[50]	(Kresken et al., 2009)	2007	Imipenem (11.1%), ciprofloxacin (27.4%), gentamycin (22.2%)	Tigecycline (MIC ≤ 0.25/1 µg/mL)	ND	ND
[16]	(Kohlenberg et al., 2009)	18 Jun till 31 Aug. 2006	Carbapenem-resistant, penicillins, cephalosporins, ciprofloxacin, gentamicin, tobramycin, tigecycline (4 mg/L), imipenem and meropenem (MIC ≥ 32 mg/L)	Colistin (MIC ≤ 0.5 mg/L) and amikacin (MIC 4–8 mg/L). Two isolates susceptible to imipenem	*bla*OXA-23-like, *bla*OXA-58-like intrinsic *bla*OXA-51	IC-2
[51]	(Rodloff et al., 2008)	Jan. 2004 till Aug. 2006	ND	Tigecycline (MIC ≤ 1 mg/L), >80% for amikacin, cefepime, ceftazidime, imipenem (MIC 1 mg/L), levofloxacin, minocycline, piperacillin-tazobactam.	ND	ND
[52]	(Weyrich et al., 2006)	ND	MDR, penicillins, cephalosporins, aminoglycosides, fluoroquinolones, rifampicin, tetracyclines, oxazolidinones, macrolides and carbapenems (imipenem and imipenem/EDTA	Colistin	Metallo-ß-lactamase	ND
[53]	(Schulte et al., 2005)	ND	Carbapenems, meropenem, penicillins, cephalosporins, aminoglycosides, quinolones	ND	Metallo-ß-lactamase	ND
[54]	(Brauers et al., 2005)	ND	MIC ≥ 64 mg/L in amoxicillin-clavulanate combinations, piperacillin-tazobactam and cefotaxime	Ampicillin-sulbactam and piperacillin-sulbactam (MIC ≤ 0.06 mg/L), meropenem (MIC ≤ 1 mg/L)	ND	ND
[14]	(Borgmann et al., 2004)	Feb. until Mar. 2003	MDR, gentamicin, aztreonam, piperacillin, piperacillin/ tazobactam, cefuroxime, cefotaxime, ceftazidime, cefepime, meropenem, tetracycline, levofloxacin, ciprofloxacin and fosfomycin	Tobramycin, amikacin and colistin	Metallo-ß-lactamase	ND
[55]	(Higgins et al., 2004b)	1991–1992	Ciprofloxacin, ofloxacin, meropenem, netilmicin, tetracycline, tobramycin, clarithromycin	ND	*gyr*A and *par*C mutation, up-regulation of *ade*B gene	ND
[56]	(Frank et al., 2003)	ND	ND	Piperacillin-sulbactam	ND	ND
[57]	(Andermahr et al., 2002)	Jun. 1996 till Jun 2001	ND	ND	ND	ND
[58]	(Heinemann et al., 2000)	Jul. 1990 till Dec. 1998	Novel quinolones superior to ciprofloxacin	Clinafloxacin most active	ND	ND

ND: not determined, MDR: Multidrug-resistant, MIC: minimum inhibitory concentrations, ST/IC: sequence type and international clone.
